# Tunisian Milk Thistle: An Investigation of the Chemical Composition and the Characterization of Its Cold-Pressed Seed Oils

**DOI:** 10.3390/ijms18122582

**Published:** 2017-12-02

**Authors:** Wiem Meddeb, Leila Rezig, Manef Abderrabba, Gérard Lizard, Mondher Mejri

**Affiliations:** 1 Laboratory of Materials, Molecules and Applications (LMMA), Preparatory Institute of Scientific and Technical Studies (IPEST), University of Carthage, La Marsa, 2070 Tunis, Tunisia; abderrabbamanef@gmail.com (M.A.); mondhermejri54@gmail.com (M.M.); 2 Faculty of Science of Bizerte, Zarzouna, 7021 Bizerte, Tunisia; 3 Biochemistry of the Peroxisome, Inflammation and Lipid Metabolism’ EA 7270, Université de Bourgogne Franche-Comté, Inserm, 21000 Dijon, France; gerard.lizard@u-bourgogne.fr; 4 Food Conservation and Valorization Laboratory, High Institute of Food Industries, 58 Avenue Alain Savary, El Khadra City, 1003 Tunis , Tunisia; l_rezig@yahoo.fr

**Keywords:** milk thistle seed oil, fatty acids, phenolic acids, tocopherols, differential scanning calorimetry

## Abstract

In this study, milk thistle seeds growing in different areas in Tunisia were cold pressed and the extracted oils were examined for their chemical and antioxidant properties. The major fatty acids were linoleic acid (C18:2) (57.0%, 60.0%, and 60.3% for the milk thistle seed oils native to Bizerte, Zaghouan and Sousse, respectively) and oleic acid (C18:1) (15.5%, 21.5%, and 22.4% for the milk thistle seed oils originating from Bizerte, Zaghouan and Sousse, respectively). High performance liquid chromatography (HPLC) analysis showed the richness of the milk thistle seed oils (MTSO) in α-tocopherol. The highest content was recorded for that of the region of Zaghouan (286.22 mg/kg). The total phenolic contents (TPC) of Zaghouan, Bizerte, and Sousse were 1.59, 8.12, and 4.73 Gallic Acid Equivalent (GAE) mg/g, respectively. Three phenolic acids were also identified (vanillic, *p*-coumaric, and silybine), with a predominance of the vanillic acid. The highest value was recorded for the Zaghouan milk thistle seed oil (83 mg/100 g). Differences in outcomes between regions may be due to climatic differences in areas. Zaghouan’s cold-pressed milk thistle seed oil had a better quality than those of Bizerte and Sousse, and can be considered as a valuable source for new multi-purpose products or by-products for industrial, cosmetic, and pharmaceutical utilization.

## 1. Introduction

Commonly known as Mary thistle or milk thistle, *Silybum marianum* L. Gaertn is an extensively distributed herbaceous plant of the Asteraceae family. It is an annual or biennial plant native to the Mediterranean, North Africa and the Middle East [1,2,3]. It grows wild but can also be cultivated [3]. Milk thistle grows abundantly in the wild in Tunisia. It has long been recognized as a remedy for bile and liver diseases [4,5]. Similarly, milk thistle is commercially cultivated for its remarkable medicinal values [6]. It has been reported that polyphenols existing in milk thistle seeds have antioxidant, anti-inflammatory, hypolipidemic, and anti-carcinogenic properties, all at once [7,8,9,10]. Milk thistle seed oil has also been documented as a potential natural source of vitamin E (fat-soluble vitamin covering a set of eight organic molecules, four tocopherols, and four tocotrienols) [11,12], and has often been recommended as a favorable edible oil [13]. In recent years, several studies were undertaken for better demonstrating the benefits of milk thistle seed oil. However, to the best of our knowledge, the composition and the physicochemical properties of milk thistle seed oil native to North Africa have never been explored before. The determination of both the physicochemical properties and the oxidative stability would certainly contribute to the valorization of the potential of milk thistle in cosmetic, pharmaceutical, and/or food industries. The present study aims at identifying the physico-chemical properties of *Silybum marianum* L. seed oils that are extracted by cold pressing and originating from different geographical areas in Tunisia. This may well contribute to the use of *Silybum marianum* L. seed oil as a novel product.

## 2. Results and Discussion

### 2.1. Chemical Analysis of Milk Thistle Seed Oils

Table 1 shows the physicochemical properties of the milk thistle seed oils that are native to Bizerte, Zaghouan, and Sousse. The specific gravitational values were 0.91, 0.91and 0.91 for milk thistle seed oils originating from Bizerte, Zaghouan, and Sousse, respectively. Those values are comparable to those that were found by Parry et al. [14], but higher than those reported by Bahl et al. [15] on an Indian SMB-5 genotype of *Sylibum marianum* L. seed oil. The iodine values of milk thistle seed oils also correspond to the iodine values that were reported by Khan et al. [16], yet higher than those reported by Bahl et al. [15]. The High iodine values of milk thistle seed oils are due to their abundance in unsaturated fatty acids (Table 2). This indicates that the seed oils are convenient for edible and/or drying oil purposes [17].

Note that the acid and peroxide values of the various milk thistle seed oils were very low. These values were even lower than those determined by the Codex Alimentarius Commission [18], which specified a permitted maximum peroxide level of no more than 10 meq of peroxide oxygen/kg oil for vegetable oils. These results are in accordance with those reported by Khan et al. and Ahmed et al. [16,19]. The lower acidity of milk thistle seed oils indicates that they are edible and might have a long shelf life [20,21]. In fact, the higher acidity index is related to the presence of Free Fatty acids (FFAs) occurring during enzymatic hydrolysis by lipases. It is worth noting that FFAs are more susceptible to oxidation than the fatty acids that are present in the triacylglycerol molecules. Hence, the higher is the level of FFAs in the seed oil, the higher is the risk of oxidation. Such a phenomenon would result in oil rancidity, which would negatively affect the oils’ sensory properties.

The saponification values of the milk thistle seed oils native to Bizerte, Zaghouan and Sousse were recorded as 205.16 mg KOH/g of oil, 194.72 mg KOH/g of oil, and 128.08 mg KOH/g of oil, respectively. The saponification value of the Bizerte milk thistle seed oil was higher than those reported by Ahmed et al. and Khan et al. [16,19]. The saponification value of the Zaghouan milk thistle seed oil, however, was in compliance with those found by Bahl et al., Ahmed et al. and Khan et al. [15,16,19].

High saponification values testify to the presence of high contents of high molecular weight triacylglycerols, which are useful for soap production [16]. Milk thistle seed oils thus show a high content in unsaponifiable matters and were within the same range of that reported by Ahmed et al. [19]. Specific extinction coefficients, which were calculated on the basis of absorbances at 232 and at 270 nm, indicate that the milk thistle seed oils contain primary (hydroperoxides) and secondary oxidation products. Variation in the characteristics of the milk thistle seed oils may be due to variations in the cultivation climate of the studied plants.

### 2.2. Fatty Acid Composition

The fatty acid composition of milk thistle seed oils is illustrated in Table 2. Eight fatty acids were detected among which three were unsaturated. The milk thistle seed oils native to Zaghouan and Sousse have higher amounts of unsaturated fatty acids. Among the unsaturated fatty acids, linoleic acid is the most abundant and account for 57.0%, 60.0%, and 60.3% for the milk thistle seed oils native to Bizerte, Zaghouan, and Sousse, respectively. The oleic acid accounted for 15.5%, 21.5%, and 22.4% for *Silybum marianum* L. seed oils from Bizerte, Zaghouan, and Sousse, respectively. These values are close to those obtained by Dabbour et al., Fathi-Achachlouei and Azadmard-Damirchi, Hasanloo et al., Ahmed et al., Khan et al., El-Mallah et al. and Parry et al. [2,13,14,16,19,22,23].

The large amounts of linoleic acid make the milk thistle seed oils more susceptible to oxidation.

### 2.3. Oxidative Stability

Results of the Rancimat test are shown in Table 1. The stability of the milk thistle seed oil expressed as the oxidation induction time was 5.83, 8.75 and 4.55 h for the seed oils that are native to Bizerte, Zaghouan, and Sousse, respectively. The oxidative stability was lower than those reported by Parry et al. (13.3 h) [14] and Dabbour et al. [22] (55.7 h) on *Silybum marianum* L. seed oils operating at 80 °C with an air flow of 7 and 20 L/h, respectively. Lower values of the oxidative stability of *Silybum marianum* L. seed oils native to different Tunisian regions may be justified by differences in the operating conditions (120 °C with an air flow of 20 L/h), and by the high contents of MUFA and PUFA (Table 2). A linear regression based on the oleic/linoleic ratio and the contents of phenols and tocopherols, in virgin olive oil, showed a good correlation with the oxidative stability measured by Rancimat. The contribution of phenolic and orthophenolic compounds in the oxidation stability of the olive oil was about 51%, 24% for fatty acids and, to fewer percentages α-Tocopherols, carotenoids, and chlorophylls [24].

### 2.4. Total Phenolic Content (TPC)

TPC of *Silybum marianum* L. seed oils ranged from 3.59 to 8.12 mg GAE/g (Table 2). These values are higher than those reported by Parry et al. and Dabbour et al. [14,22] on cold pressed milk thistle seed oils (3.07 mg GAE/g and 1.16 mg GAE/g, respectively). TPC of *Silybum marianum* L. seed oils native to Tunisia are also higher than other cold pressed seed oils such as parsley, cardamom, mullein, onion, and roasted pumpkin showing a TPC ranging from 0.98 to 3.35 mg GAE/g oil [14]. TPC of Tunisian *Silybum marianum* L. seed oils are also higher than those reported on Red Raspberry, Bluebarry, Boysenberry, and Marionberry, ranging from 0.09 mg GAE/g oil to 1 mg GAE/g oil for 80% MeOH extracts and from 1.49 mg GAE/g oil to 2 mg GAE/g oil for 100% MeOH extracts [25]. Statistical analysis revealed significant differences between TPC of *Silybum marianum* L. seed oils originating from Sousse, Bizerte, and Zaghouan. The variation in TPC may be due to the differences in cultivation climate and crop growing conditions, such as soil, irrigation, and temperature. It is noteworthy to pinpoint the linear correlation between oxidation time and TPC of *Sylibum marianum* L. seed oils. The milk thistle seed oil native to Zaghouan recorded the highest amount of TPC (8.12 mg GAE/g) and showed in the same way the highest oxidative stability (8.57 h). Such a result confirmed the findings of Aparicio et al. [24] on the significant contribution of phenolic compounds (51%) to the oxidative stability.

### 2.5. Phenolic Acids and Flavonoids

Phenolic compounds are part of the unsaponifiable matter and are known as minor oils constituents. These compounds play a major role thanks to their particular features, such as flavor, shelf life, and resistance against oxidation. A list of the determined phenolic acids along with their concentrations is illustrated in Table 2. Obviously, *Silybum marianum* L. seed oils are not rich in phenolics. Only *p*-coumaric acid and vanillic acid were identified. The amount of *p*-coumaric acid ranged between 0.26 mg/100 g for the Zaghouan milk thistle seed oil and 0.9 mg/100 g for that of Sousse. Vanillic acid was not detected in the milk thistle seed oil originating from Bizerte. However, its concentration reached 40.2 and 83 mg/100 g for the milk thistle seed oil native to Sousse and Zaghouan, respectively. The variability in phenolic acid contents could be attributed to cultivation climates and edaphic variables. *p*-Coumaric acid and vanillic acid were not detected in the achenes of 15 milk thistle cultivars studied by Lucini et al. [26] and originating among the main European-producing countries. They reported the presence of caffeic, ferulic, and chlorogenic acids with concentrations ranging between 2.2 and 33.6 mg/kg, 9.7 and 26.5 mg/kg and 126.5 and 395.3 mg/kg, respectively. Silybin is one of the three main compounds of the silymarin complex, also known as flavonolignans, which are the most present flavonoids in milk thistle seeds. When compared to silydianin and silychristin, silybin is the component with the highest biological activity and represents 50–70% of the silymarine complex [26]. Note that silybine was not identified in the milk thistle seed oil originating from Sousse. In *Silybum marianum* L. seed oils native to Zaghouan and Bizerte, silybum was detected, but its concentration was not determined. Ҫelik and Gürü [27] reported a silybin concentration of 2.29 mg/g seeds for silybin A and 1.92 mg/g seeds for silybin B after milk thistle seed oil extraction by supercritical CO_2_. These values are lower than those that were reported by Lucini et al. [26] on 15 genotypes of *Silybum marianum* L. achenes, where silybin content ranged between 3826 mg/kg and 9499 mg/kg. Differences in silymarin contents are attributed to a number of factors such as genotypes and environmental conditions [28,29,30].

### 2.6. Tocopherols

Tocopherols are by far important biological antioxidants. Alpha-tocopherol, or vitamin E, prevents oxidation of body lipids including polyunsaturated fatty acids and lipid components of cells and organelle membranes. Tocopherol has also been associated with the reduction of heart diseases, the delay of Alzheimer’s disease and cancer prevention [21].

Table 2 shows tocopherol composition and contents in the three cold pressed *Silybum marianum* L. seed oils samples originating from Bizerte, Sousse, and Zaghouan. Statistical analyses have revealed significant differences in the tocopherol content among milk thistle seed oils. The results have demonstrated that the ripening conditions significantly affect the content of the tocopherol being analysed. α- and β-Tocopherols were the only ones detected in cold pressed *Silybum marianum* L. seed oil native to Bizerte and accounted for 47.65 mg/kg and 1.91 mg/kg, respectively.

In the cold pressed *Silybum marianum* L. seed oils native to Zaghouan and Sousse, the four tocopherols were detected with a predominance of the α-Tocopherol which accounted for 98.92% and 88.59% in the Zaghouan and Sousse milk thistle seed oils, respectively. The α-Tocopherol amounts in Zaghouan and Sousse milk thistle seed oils are in the same range of those that were reported by El-Mallah et al. [13] for the *Silybum marianum* L. seed oil extracted by chloroform-methanol (84.5%) and those reported by Fathi-Achachlouei and Azadmard-Damirchi [2] for the Iranian Barak castle and CN-milk thistle seed oil varieties extracted by hexane/isopropanol (84.9% and 86.36%, respectively). Nevertheless, the α-Tocopherol amount was higher than that cited by Parry et al. [14] (78.78%) on cold pressed milk thistle seed oil and by Hasanlo et al. [23] (69.02%) on *Silybum marianum* L. seed oil extracted by petroleum ether in Soxhlet apparatus. The α-Tocopherol content in the Sousse milk thistle seed oil was followed by γ-Tocopherol, β-Tocopherol and the δ-Tocopherol. β- and γ-Tocopherol amounts were higher than those that were observed in the Zaghouan milk thistle seed oil. According to O’Brien [31], the α-isomer is the most biologically active, whereas the γ-Tocopherol is perceived as the best antioxidant as it inhibits lipid oxidation by stabilizing hydroperoxy and other free radicals.

In short, the cold pressed milk thistle seed oil native to Zaghouan is an ideal dietary source of total α-Tocopherol, whereas that of Sousse may well serve as a dietary source of γ-Tocopherol.

### 2.7. Thermal Behavior

**Figure 1:**
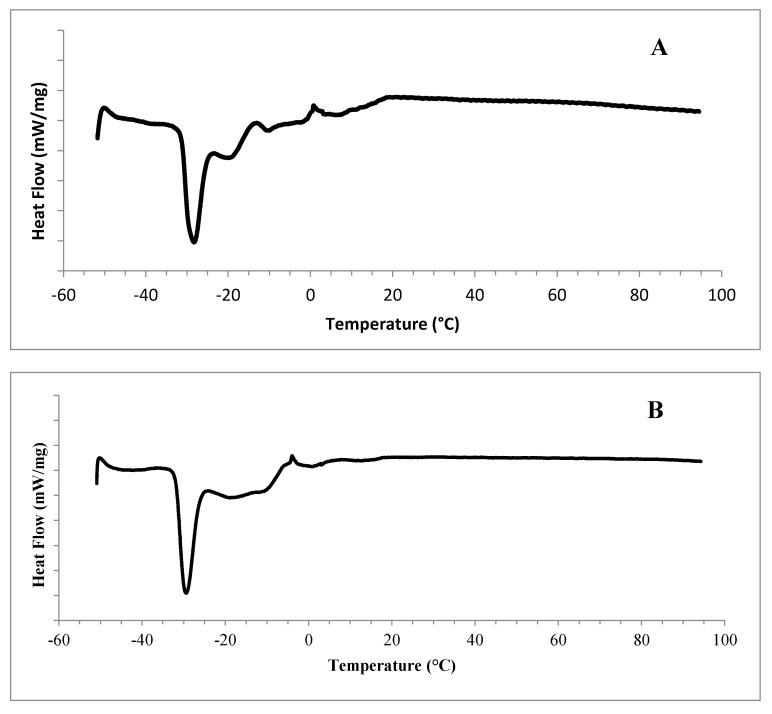

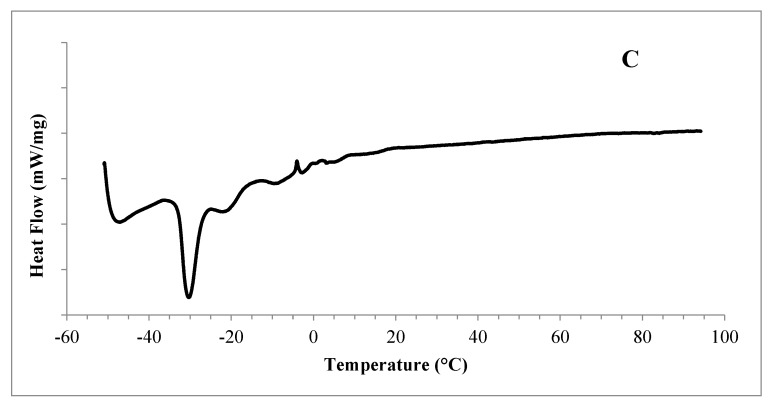
Melting thermograms of *Silybum marianum* L. seed oils native to Zaghouan (**A**), Sousse (**B**), and Bizerte (**C**).

The melting curves of *Silybum marianum* L. seed oils native to Zaghouan, Sousse and Bizerte are illustrated in Figure 1. When heated from −50 to 90 °C, the Zaghouan *Silybum marianum* L. seed oil showed two shoulder merging peaks that occurred at −48.55 and −40.55 °C. Yet, the first endothermic transition was observed at −44.84 °C for the Sousse milk thistle seed oil, with a typical one at −48.9 °C for the Bizerte *Silybum marianum* seed oil. These endothermic transitions could be attributed to the melting of the lowest stability polymorphic forms of triacylglycerols (TAG) (e.g., α-TAG) [32].

A distinct sharp narrow peak occurred at −29.02, −29.86 and −30.92 °C for the Zaghouan, Sousse and Bizerte seed oils, respectively. Beyond this typical and distinguishable peak, other endothermic transitions occurred, too. In fact, the Zaghouan *Silybum marianum* L. seed oil showed four transitions that occurred at −20.51, −11.91, −3.06 and 4.57 °C. Furthermore, The Sousse seed oil exhibited four shoulder merging peaks, which occurred, respectively, at −20.63, −11.57, −0.4 and 11.36 °C. However, the Bizerte seed oil showed five endothermic transitions that occurred at −22.92, −11.63, −3.3, 2.89 and 11.62 °C. All of the endothermic transitions, occurred beyond the typical one, illustrated approximately the same temperature range (the range of the transitions can be calculated as temperature difference between T_on_ and T_off_). The last transitions could be associated with the melting of the highest stability polymorphic forms of TAG, mainly monosaturated triacylglycerols (MSTAG). Yet, the disaturated triacylglycerols (DSTAG) could not be excluded. Nyam et al. [21] reported that vegetable oils with high content of saturated fatty acids (SFA) experienced DSC melting profiles at higher regions as compared to oils with a high content of unsaturated fatty acids (UFA). The complex endothermic events occurring at higher temperatures were attributed to the melting of crystallized lipids and were characterized by multiple overlapping contributions as previously observed in vegetable [33,34] and olive oils [34,35,36]. No endothermic phenomenon was illustrated beyond 12 °C. Such a feature may confirm the liquid state of the *Silybum marianum* L. seed oils at room temperature (25 °C), and consequently the absence of crystals.

### 2.8. Colour

Colour is another important characteristic for determining the visual approval of oil. The CieLab coordinates values (L*, a*, b*) of cold pressed *Silybum marianum* L. seed oils originating from Bizerte, Sousse, and Zaghouan are illustrated in Table 3. The tested seed oils differed in their colours. The Bizerte milk thistle seed oil showed a higher L* and a lower a* values when compared to those of Zaghouan and Sousse. This means that the cold pressed milk thistle seed oil native to Bizerte was lighter in colour than those of Zaghouan and Sousse. The CieLab (L*, a*, b*) values of other vegetable oils such as palm, soybean, sunflower, olive, and corn ranged from 63.4 to 69.5, 3.8 to 4.4, and 9.2 to 10.4, respectively [37]. Hence, the cold pressed milk thistle seed oils a* values were lower than those of other vegetable oils. Whereas, b* values at the exception of Zaghouan milk thistle seed oil were higher than those that were reported by Hsu and Yu [37]. This suggests the presence of yellow pigments, such as carotenoids, in milk thistle seed oils. The Bizerte *Silybum marianum* L. seed oil was marked by another colour specification: a* negative value which was markedly lower than the a* value of common vegetable oils.

## 3. Materials and Methods

### 3.1. Seed Material

**Figure 2:**
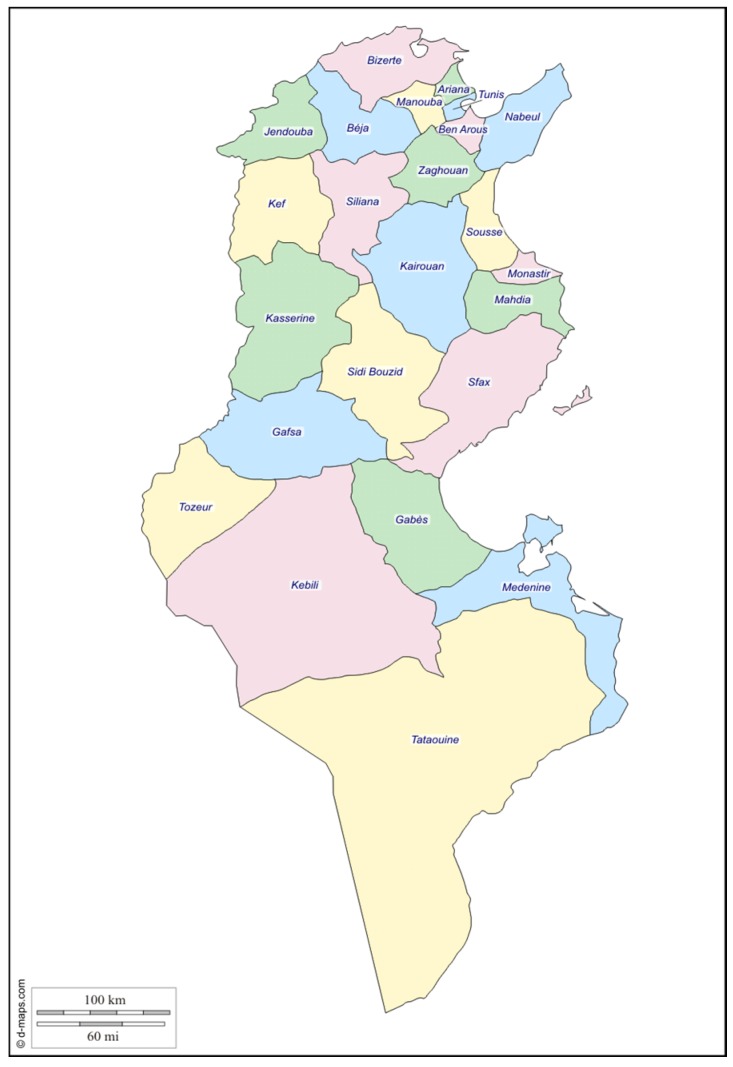
Map of Tunisia indicating the regions where milk thistle seed samples were gathered.

The present study is mainly concerned with milk thistle seeds that were gathered from three different regions in Tunisia, namely Zaghouan (latitude 36°24′32.8″; longitude 10°8′32.3″; elevation 176 m), Bizerte (latitude 37°16′27″; longitude 9°52′26″; elevation 5 m), and Sousse (latitude 35°49′31″; longitude 10°38′13″; elevation 24 m) (See Figure 2). The plant was picked both in May and June 2014, a period during which the plant reaches maturity and senescence.

### 3.2. Oil Extraction

Milk thistle (*Silybum marianum* L.) seed oil was obtained by cold pressing using an MUV2 65 vegetable oil screw press (Smir Technotour, Agadir, Morocco). This technique obviously provides a cold extraction of the solid sample oil contained within a plant hopper.

The oil extraction was carried out when the milk thistle seeds were ground and compressed under the pressure that was exerted by the conical screw rotation. The oil was forced into the perforated tube. The meal was then evacuated at the end of the shaft by a calibrated orifice that often interchangeably acts as a barrier to the flow of the meal (residual fat, nutritional value, etc.). The remaining oil flows to the centrifuge for 15 min in order to separate the oil from plant material debris. This is automatically followed by filtration. The seeds are then stored in a freezer at (−20 °C) for further analysis.

### 3.3. Proximate Analyses

#### 3.3.1. Chemical Analysis

American Oil Chemists’Society (AOCS) [38] were used for the determination of the acid value (method Cd 3d-63), peroxide value (method Cd 8-53), iodine value (method Cd 1-25), specific gravity (using a 10 mL pycnometer at 25 °C), unsaponifiable matter (method Ca 6a-40), saponification value (method Cd 3-25), and the refractive index (using Abbé refractometer at 40 °C) of the milk thistle seed oil.

Specific absorptivity values K_232_ and K_270_ were determined using a UV spectrophotometer by measuring the absorbance of a 1% milk thistle seed oil solution in cyclohexane and a path of 1 cm at 232 and 270 nm.

#### 3.3.2. Fatty Acid Composition

Fatty acids were determined by the analytical methods described in the European Parliament and the European Council in EEC regulation 2568/91 [39]. The fatty acids were converted to fatty acid methyl esters (FAMEs) before being analyzed. This is done through shaking off a solution of 0.2 g of oil and 3 mL of hexane with 0.4 mL of 2 N methanolic potassium hydroxide. The FAMEs were then analyzed in a Hewlett-Packard model 4890D Gas Chromatograph furnished with an HP-INNOWax fused silica capillary columns (Cross-Linked PEG), 30 m × 0.25 mm × 0.25 m and a flame ionization detector (FID). Inlet and detector temperatures were held at 230 °C and 250 °C, respectively. The initial oven temperature was held at 120 °C for 1 min and then it was raised to 240 °C at a rate of 4.0 °C/min for 4 min. The FAMEs injected volume was 1 µL and nitrogen (N_2_) was used as the carrier gas at 1 mL/min with a split inlet flow system at a 1:100 split ratio. Then, heptadecanoic acid C17:0 was added as an internal standard before methylation, so as to measure the amount of fatty acids. Eventually, the fatty acid contents were measured using a 4890A Hewlett-Packard integrator. The FAMEs peaks were identified by comparison with the retention times of a standard mixture. The peak areas were computed and the percentages of the FAME were obtained as area percentages by direct normalization.

#### 3.3.3. Determination of Total Phenolic Content (TPC)

One gram of each *Silybum marianum* L. seed oil was extracted with 3 mL of MeOH by vortexing at ambient temperature [25]. The MeOH extracts were collected by centrifugation. The oil residues were re-extracted twice with MeOH (3 mL × 2). The three MeOH extracts were combined and the final volume was brought to 10 mL with MeOH to obtain the testing sample solutions. The Folin-Ciocalteu (FC) reagent was used to determine the TPC of *Silybum marianum* L. seed oils, according to Yu et al. [40].

Briefly, the reaction mixture containing 250 μL of fresh FC reagent, 750 μL of 20% Na_2_CO_3_ and 3 mL of deionized water was added to 50 μL of the testing sample solution (oil extract), or to the standard to induce reaction. Absorbance was determined at 765 nm right after 2 h of reaction at ambient temperature and used to calculate the TPC in oils. Gallic acid was used as the standard for measurement. Values were given as mg gallic acid equivalent (GAE) per g of oil.

#### 3.3.4. Identification and Quantification of Phenolic Acids and Flavonoids by HPLC-MS/MS

The HPLC-MS experiments were carried out using an Agilent 1100 LC system consisting of a degasser, a binary pump, an auto sampler, and a column heater [41].

The column outlet was coupled to an Agilent MSD Ion Trap XCT mass spectrometer equipped with an ESI ion source. Data acquisition and mass spectrometric evaluation were carried out on a personal computer using CHEMCAD 6.3, a chemical process simulation software that is designed by Chemstation, Inc. (Dayton, OH, USA). For the chromatographic separation, a Knauer C-18 column (250 mm × 4.6 mm, 5 µm) was used. The column maintained at 40 °C was first held at 90% of Solvent A (1% acetic acid in water) and 10% of solvent B (1% acetic acid in methanol), followed by a step gradient from 10% B to 20% B in 4 min and a second step gradient from 20% B to 100% B in 20 min. It was later kept for 6 min with 100% B. Finally, the elution was obtained from 100% B to 20% B for 6 min. The flow rate was 400 µL/min and the injected volume was 10 µL of the mixture that contained 100 µL of milk thistle seed oil and 900 µL of ethyl acetate.

The following parameters were used throughout the MS experiment, i.e., for the electrospray ionization with negative ion polarity. The capillary voltage was set to 1.6 kV, the drying temperature to 350 °C, the nebulizer pressure to 40 psi, and the drying gas flow to 10 L/min. The maximum accumulation time was 50 ms, the scan speed was 26,000 m·Z^−1^·s^−1^ (Ultra Scan Mode) while the fragmentation time was 30 ms. Phenolic compounds were identified using a combination of high performance liquid chromatography (HPLC) with an Agilent 1100 diode array detection and liquid chromatography with electrospray ionization mass spectrometry (ESI-LC-MS) on the basis of their ultraviolet (UV) spectra, mass spectra, and by comparing the spectra with those of available authentic standards [41].

#### 3.3.5. Tocopherol Composition

Prior to the HPLC analysis, 0.5 g of the seed oil was diluted with 5 mL of hexane, and a 5 µL sample was then injected. The tocopherol composition of seed oil was determined using HPLC according to the NF EN ISO 9936 [42]. The sample was then analyzed by an HPLC (Agilent 1100, Palo Alto, CA, USA), consisting of a G1354 quaternary pump, a G1313A standard autosampler, a G 1321A fluorescence detector set at λ excitation = 295 nm and λ emission = 330 nm and a Chemstation software. A normal phase column (hypersil silica, Hewlett Packard) (250 mm × 4.6 mm × 5 µm) was used with hexane/isopropanol (99.5:0.5, *v*:*v*) as a mobile phase. The system was operated isocratically at a flow rate of 0.5 mL/min. The separations were carried out at 30 °C. The quantification was based on an external standard method and the tocopherol standards mixed in hexane solution (2 mg/mL) were prepared on the basis of the standard compounds: α-, β-, γ- and the δ-Tocopherols.

#### 3.3.6. Differential Scanning Calorimetry (DSC)

Thermal properties of milk thistle seed oil were determined using a modulated differential scanning calorimeter (DSC 2920 Modulated DSC-TA Instruments, Newcastle, DE, USA). The oil sample (2 ± 0.10 mg) was weighed directly into a DSC-pan (SFI-Aluminium, TA Instrument T11024, Newcastle, DE, USA). The seed oil was quickly cooled to −50 °C with a speed of 15 °C/min, maintained for 15 min, and then heated to 90 °C with a heating speed of 15 °C/min. The heating process was repeated twice and the DSC thermographs were recorded during the second melting. The instrument was calibrated for temperature and heat flow using eicosane (Tp = 36.8 °C, H = 247.70 J/g) and dodecane (Tp = −9.65 °C, H = 216.73 J/g). An empty DSC-pan was used as a reference [41].

#### 3.3.7. Oxidative Stability

The oxidative stability was determined with the 743 Rancimat apparatus (Metrohm Co., Basel, Switzerland), an instrument for the automatic determination of the oxidation stability of fats and oils. The stabilization level was measured by the oxidative-induction time (OIT) using 3.5 g of oil. The temperature was set at 100 °C, and the purified air flow passing through at a rate of 10 L/h. During the oxidation process, volatile acids were formed in the distilled water and were measured conductimetrically. The induction period was defined as the necessary time to reach the inflection point of the conductivity curve [43].

#### 3.3.8. CIE L* a* b* Coordinates

CieLab coordinates (L*, a* and b*) were directly read with a spectrophotometer colorimeter (Trintometer, Lovibond, Cambridge, UK). In this coordinate system, the L* value is a measure of lightness, ranging from 0 (black) to 100 (white). The a* value, represents the red/green axis, and varies from −100 (greenness) to +100 (redness) and the b* value represents the yellow/blue axis and ranges from −100 (blueness) to +100 (yellowness).

#### 3.3.9. Data Analysis

All of the analyses were performed in triplicate and the results were expressed as mean values ± standard deviations (SD). The data were subjected to statistical analyses using a statistical program package [44]. The one-way analysis of variance (ANOVA) followed by the Tukey’s test was employed and the differences between individual means and each means used were deemed to be significant at *p* < 0.05.

## 4. Conclusions

This study has fairly demonstrated that *Silybum marianum* L. seeds are a rich source of many key nutrients that seem to have a very positive effect on human health. Similarly, phenolics and tocopherols are rich in antioxidants and may provide a high protection against oxidative stress. Along with the relatively good shelf life and few other favorable characteristics, the milk thistle seed oil is an ideal ingredient for both pharmaceutic and cosmetic applications.

The results confirm, to a certain extent, that the significant discrepancy in the bioactive compounds and the oxidative stability of the different seed oils might be attributed to the different growing regions of milk thistle, to the different climates, and the different soil compositions. In the light of its chemical profile, the Zaghouan’s milk thistle seed oil proves to have a better quality in comparison to those of Bizerte and Sousse.

From among the targeted phenolic compounds, silybin, a flavonolignan coumpound of silymarin complex, was detected only in the *Silybum marianum* L. seed oils of Bizerte and Zaghouan. Since silybin is known as the component with the highest biological activity, further studies are required in order to characterize the phenolic profile and the antioxidant activity of the milk thistle seeds from other regions of Tunisia. Such an investigation could demonstrate the influence of the milk thistle’s environmental background on the chemical and antioxidant profiles of its achenes.

## Figures and Tables

**Table 1:** Physicochemical characterization of cold pressed milk thistle seed oils originating from Tunisia.

Region	Bizerte	Zaghouan	Sousse
Refractive index (40 °C)	1.47 ± 0.12 ^a^	1.46 ± 0.11 ^b^	1.47 ± 0.13 ^a^
Specific gravity (25 °C)	0.91 ± 0.08 ^a^	0.91 ± 0.08 ^a^	0.91 ± 0.08 ^a^
Acid value (mg KOH/g oil)	5.48 ± 0.51 ^c^	7.59 ± 0.61 ^b^	8.34 ± 0.77 ^a^
Peroxide value (meq O_2_/kg oil)	2.83 ± 0.24 ^c^	3 ± 0.27 ^b^	4.20 ± 0.38 ^a^
Iodine value (g I_2_/100 g oil)	112.41 ± 11.02 ^c^	118.32 ± 10.84 ^a^	116.10 ± 10.46 ^b^
Saponification value (mg KOH/g oil)	205.16 ± 18.75 ^a^	194.72 ± 18.42 ^b^	128.08 ±11.59 ^c^
Unsaponifiable matter (%)	1.57 ± 0.465 ^c^	4.96 ± 0.24 ^b^	5.84 ± 0.17 ^a^
Oil stability index (h)	5.83 ± 0.48 ^b^	8.75 ± 0.77 ^a^	4.55 ± 0.35 ^c^
K_232_	2.17 ± 0.20 ^a^	1.89 ± 0.15 ^c^	2.02 ± 0.21 ^b^
K_270_	0.30 ± 0.02 ^b^	0.24 ± 0.02 ^c^	0.45 ± 0.03 ^a^

Values with different letters are significantly different *p* < 0.05. Values are means ± Standard Deviations (SD) of three determinations.

**Table 2:** Fatty acid (%) composition, tocopherol content (mg/kg oil), total phenolic content (mg GAE/g) and phenolic acids and flavonoids (mg/100 g) of milk thistle seed oils originating from Tunisia.

Region	Bizerte	Zaghouan	Sousse	
Fatty acids	Composition			
Palmitic (C16:0)	7.00 ± 0.68 ^b^	5.50 ± 0.41 ^c^	11.40 ± 1.21 ^a^	
Stearic (C18:0)	4.50 ± 0.35 ^b^	4.75 ± 0.39 ^a^	2.90 ± 0.24 ^c^	
Oleic (C18:1)	15.50 ± 1.48 ^c^	21.50 ± 2.11 ^b^	22.40 ± 2.21 ^a^	
Linoleic (C18:2)	57.00 ± 6.28 ^c^	60.00 ± 5.94 ^b^	60.30 ± 6.58 ^a^	
Arachidic (C20:0)	2.50 ± 0.19 ^b^	2.90 ± 0.20 ^a^	1.80 ± 0.17 ^c^	
Eicosenoic acid (C20:1)	0.72 ± 0.06 ^b^	0.85 ± 0.07 ^a^	tr.	
Behenic acid (C22:0)	2.25 ± 0.21 ^b^	2.50 ± 0.20 ^a^	0.92 ± 0.08 ^c^	
Lignoceric acid (C24:0)	0.55 ± 0.04 ^c^	0.60 ± 0.05 ^b^	0.92 ± 0.07 ^a^	
SAFA	16.81 ± 1.45 ^b^	16.26 ± 1.58 ^c^	17.95 ± 1.84 ^a^	
MUFA	16.23 ± 1.28 ^c^	22.36 ± 3.12 ^b^	22.41 ± 2.69 ^a^	
PUFA	57.00 ± 4.58 ^b^	60.00 ± 5.73 ^ab^	60.31 ± 6.12 ^a^	
Tocopherol	Composition			
α	47.65 ± 3.54 ^c^	286.22 ± 25.49 ^a^	278.47 ± 24.64 ^b^	
β	1.91 ± 0.21 ^c^	3.58 ± 0.37 ^b^	6.66 ± 0.74 ^a^	
γ	0.00	14.24 ± 1.25 ^b^	23.94 ± 2.14 ^a^	
δ	0.00	14.24 ± 1.22 ^a^	5.23 ± 0.61 ^b^	
Total	49.57 ± 5.11 ^c^	318.29 ± 28.45 ^a^	314.31 ± 30.77 ^b^	
Total Phenolic Content	Composition			
	3.59 ± 0.14 ^c^	8.12 ± 0.75 ^a^	4.73 ± 0.39 ^b^	
Phenolic acids and flavonoids	Composition			Fragments MS/MS (*m*/*z*)
p-coumaric acid	0.34 ± 0.16 ^b^	0.26 ± 0.07 ^c^	0.90 ± 0.03 ^a^	148.7/130.8/119.9/84.7
vanillic acid	ni	83 ± 2.4 ^a^	40.20 ± 1.8 ^b^	159.1/139.7/123.8/97.8/75.9
Sylibine	*	*	ni	452.6/434.6/354.8/300.7/282.7/256.8/214.8/186.8

Values with different letters in the same raw are significantly different *p* < 0.05. SAFA: saturated fatty acids; MUFA: monounsaturated fatty acids; PUFA: polyunsaturated fatty acids. tr.: trace amounts (less than 0.2%). ni: not identified. *: Compound detected but concentration in sample not determined. Values are means ± SD of three determinations.

**Table 3:** CieLab coordinates (L*, a*, b*) of cold pressed milk thistle seed oils originating from Tunisia.

	L*	a*	b*
**Bizerte**	66.07 ± 6.25 ^a^	−0.85 ± 0.21 ^c^	12.10 ± 1.11 ^b^
**Zaghouan**	59.93 ± 2.763 ^a^	0.35 ± 0.02 ^c^	5.78 ± 0.62 ^b^
**Sousse**	41.94 ± 4.11 ^a^	1.53 ± 0.14 ^c^	14.95 ± 1.32 ^b^

Values with different letters in the same column are significantly different *p* < 0.05. Values are means ± SD of three determinations.
